# Dietary Interventions with Polyphenols in Osteoarthritis: A Systematic Review Directed from the Preclinical Data to Randomized Clinical Studies

**DOI:** 10.3390/nu13051420

**Published:** 2021-04-23

**Authors:** Evdokia Valsamidou, Aristea Gioxari, Charalampia Amerikanou, Panagiotis Zoumpoulakis, George Skarpas, Andriana C. Kaliora

**Affiliations:** 1 Department of Nutrition and Dietetics, School of Health Science and Education, Harokopio University, 18345 Athens, Greece; evdokiavalsamidou@gmail.com (E.V.); arisgiox@gmail.com (A.G.); amerikanou@windowslive.com (C.A.); 2 Qualia Pharma, Ν. Kifissia, 14564 Attiki, Greece; pzoump@uniwa.gr; 3 Department of Food Science and Technology, University of West Attica, Ag. Spyridonos, Egaleo, 12243 Athens, Greece; 4 Hellenic Open University/Sports Injuries & Regenarative Medicine Orthopaedic Clinic at “MITERA” Hospital, Marousi, 15123 Attiki, Greece; skarpasg@gmail.com

**Keywords:** anti-inflammatory, antioxidant, polyphenols, osteoarthritis, synergy

## Abstract

Osteoarthritis (OA) is the most common form of arthritis and a major cause of limited functionality and thus a decrease in the quality of life of the inflicted. Given the fact that the existing pharmacological treatments lack disease-modifying properties and their use entails significant side effects, nutraceuticals with bioactive compounds constitute an interesting field of research. Polyphenols are plant-derived molecules with established anti-inflammatory and antioxidant properties that have been extensively evaluated in clinical settings and preclinical models in OA. As more knowledge is gained in the research field, an interesting approach in the management of OA is the additive and/or synergistic effects that polyphenols may have in an optimized supplement. Therefore, the aim of this review was to summarize the recent literature regarding the use of combined polyphenols in the management of OA. For that purpose, a PubMed literature survey was conducted with a focus on some preclinical osteoarthritis models and randomized clinical trials on patients with osteoarthritis from 2018 to 2021 which have evaluated the effect of combinations of polyphenol-rich extracts and purified polyphenol constituents. Data indicate that combined polyphenols may be promising for the treatment of osteoarthritis in the future, but more clinical trials with novel approaches in the identification of the in-between relationship of such constituents are needed.

## 1. Introduction

Osteoarthritis (OA) is a chronic degenerative joint disease characterized by progressive destruction of the articular cartilage. It affects joints of the body with a greater range of motion, referred to as the diarthrodial joints. Structurally, a diarthrosis comprises, from the inside out, of (a) articular cartilage, which lines the end of the bones; (b) synovial fluid, a lubricating media between two bones; and (c) the joint capsule, which consists of the outer fibrous capsule and the synovial membrane. OA is the most common arthritic malady globally. As expected, the burden of this disease on the health care system is increasing due to the aging of the population [1].

According to Osteoarthritis Research Society International (OARSI), OA “manifests first as a molecular derangement (abnormal joint tissue metabolism) followed by anatomic, and/or physiologic derangements (characterized by cartilage degradation, bone remodeling, osteophyte formation, joint inflammation and loss of normal joint function)” [2]. These three domains—molecular, anatomic, and physiologic disease elements—culminate to illustrate the multifaceted pathophysiology of the disease.

Structural alterations are manifested clinically as cardinal symptoms of OA such as pain, crepitus, stiffness, loss of function, erythema and edema. However, the complexity of OA pathophysiology translates to heterogeneous phenotypes that call for different approaches [3]. Delineation of the related phenotypes is challenging and difficult to address and incorporate in the study design of clinical interventions, albeit a meaningful proposition given the poor trial outcomes and limited effective treatment options for OA [4].

Revised recommendations by the American College of Rheumatology (ACR) for the current treatment approach in knee OA comprise of exercise, weight loss, topical and oral nonsteroidal anti-inflammatory drugs (NSAIDs), and intraarticular glucocorticoid injections [5]. Hyaluronic acid injections are also applied as lubricating agents, while autologous mesenchymal stem cell (MSC) treatment constitutes a rather recent and effective alternative for the treatment of knee OA [6].

In view of the urgent need for more effective treatments for OA with fewer side effects, several dietary compounds have been examined for their therapeutic potential. OA is a noninfectious civilization disease, and various dietary interventions may predict or slow its progression. Among different nutrients, non-nutrient components, e.g., polyphenols (which are important constituents of a human diet), may be beneficial in the prevention of OA due to their antioxidant and anti-inflammatory properties [7]. As their mechanisms of action in OA are being elucidated, an opportunity arises for their combined use in clinical trials. With that in mind, a review was conducted with the aim of recording recent literature that has evaluated the additive and/or synergistic effects of polyphenols in preclinical models or clinical trials in the management of OA.

## 2. Pathophysiology of OA

Articular cartilage is avascular, alymphatic, and thus devoid of an intrinsic repair system. Embedded in the extracellular matrix (ECM) of the cartilage, the only resident cell of this tissue the articular chondrocyte (AC) resides. Looking closely at the molecular derangements that predate the anatomic/physiologic hallmarks of OA, it is evident that ACs play a central role in the pathophysiology of OA [8]. Under normal metabolic stimulus, ACs maintain a delicate balance between cartilage synthesis and degradation. However, in the case of OA, low-grade inflammation is established [9] which favors catabolism mediated by enzymes such as matrix metalloproteinases (MMPs) and aggrecanases, a disintegrin and metalloprotease with thrombospondin motifs (ADAMTS). In this unfavorable environment, ACs proliferate and expand, creating clusters. These processes lead to cell apoptosis and endochondral ossification [10]. 

Low-grade inflammation and oxidative stress (OS) are the two deleterious entities that supply the vicious circle of OA. 

Upon activation of innate immunity through the recognition of damage associated molecular patterns (DAMPs) by pattern recognition receptors (PRRs), inflammatory signaling pathways are activated [11], with cytokines and chemokines being the end-products. Different cells bear different PRRs and promote different signal transduction pathways. For instance, toll-like receptors (TLR) activate the myeloid differentiation factor 88 (MyD88)-dependent signaling pathway or the toll/interferon response factor (TRIF)-dependent signaling pathway [12], and NOD-like receptors (NLRs) activate pathways of transcription factors, i.e., the nuclear factor kappa-Β (NF-κΒ) [13] and the activator protein 1 (AP-1). 

Macrophages of the synovium and complement cascade are also implicated in OA pathology. Interestingly, macrophages can adopt different phenotypes depending on the microenvironment stimuli. During the inflammatory stage, they release proinflammatory mediators such as IL-1β and are characterized as classically activated macrophages/M1. When they secret growth factors, they attain the alternatively activated macrophage/M2 phenotype. At the onset of OA, activated macrophages undergo reprogramming and are polarized to the M1 phenotype, sustaining, by extension, not only synovitis but also the dysregulation of signaling pathways in ACs that control the integrity of the cartilage [14]. Adaptive immunity can likewise be involved in the dynamic polarization of the macrophages via the Th2 cells and the secretion of either interleukin 4 (M2 phenotype) or interferon gamma (Μ1 phenotype) [15]. Nevertheless, the exact interplay between the cells of the adaptive immunity and the OA environment has not been fully elucidated yet [16].

Another example of a physiological cellular function turning against the cells in deleterious environments is the involvement of reactive oxygen species (ROS) in signal transmission, which inhibits matrix synthesis and promotes matrix degradation and cell apoptosis/senescence [17]. Under normal function of homeostatic mechanisms, a low number of ROS are generated by ACs that play an integral role in cellular functions [18]. However, when the levels of nitric oxide (NO) synthetized by NO synthase enzymes and superoxide anion (O_2_^−^) produced by the NADPH oxidase complex exceed the antioxidant capacities of the microenvironment, OS is established, further disrupting the cartilage homeostasis [19].

## 3. Polyphenols

Polyphenols are a class of phytochemicals and secondary metabolites of plants with high antioxidant properties. Their structure comprises of at least two phenyl rings and one or more hydroxyl substituents. There are four classes of polyphenols, namely flavonoids, phenolic acids, stilbenes, and lignans. Flavonoids can be further categorized into flavanols, flavanones, flavones, flavonols, isoflavones, and anthocyanins. A well-established body of literature confirms their potent contribution in OA treatment [20,21,22]. 

Preclinical evaluation of the chondroprotective effects of polyphenols, such as epigallocatechin gallate (EGCG), curcumin, carnosol, hydroxytyrosol (HT), and resveratrol, has shown that these constituents can improve clinical indices and decrease catabolic enzymes, inflammatory cytokines, and oxidative markers [23,24]. 

At the clinical level, dietary polyphenols have been evaluated in randomized controlled trials (RCTs) such as freeze-dried strawberry powder [25], freeze-dried blueberry powder [26], pomegranate juice [27], tea rich in rosmarinic acid (RosA) [28], and tart cherry juice [29]. Whereas these interventions have reported several beneficial effects, the fact that serum and plasma biomarkers do not always correlate with statistically significant improvement of pain and quality of life highlights the complexity of OA manifestation. Furthermore, lack of uniformity in the primary endpoints set, variation in study duration, and heterogeneity of the administered mixtures do not allow for the definition of their therapeutic attributes. 

Extracts from medicinal plants have been extensively investigated for different morbidities, especially in the context of Ayurvedic medicine. Panahi and colleagues (2016) [30] explored the effects of *Elaeagnus angustifolia* extract, which is rich in kaempferol, ferulic acid, and coumaric acid, in knee OA. Patients were enrolled in a 7-week intervention and randomized to receive either a high dose of the extract or a low dose or ibuprofen. The Western Ontario and McMaster Universities Osteoarthritis Index (WOMAC), the Visual Analog Scale (VAS), Lequesne’s Pain-Function Index (LPFI), and the patient’s global assessment (PGA) index decreased significantly in all groups but the effect between groups did not reach statistical significance. Another trial [31] evaluated the extract of *Artemisia annua,* formulated as a dietary supplement, in 42 patients randomized in three groups receiving a low dose of the extract, a high dose of the extract, or a placebo for 3 months. WOMAC and VAS were significantly reduced only in the low-dose group and the extract was well tolerated.

## 4. Synergy/Pharmaceuticals

A well-known study by Chou et al. published in the mid 1980s [32] focused on the standardization of a theory for quantifying the synergistic interactions between drugs. Currently, this theory is timely as it regards naturally occurring substances with therapeutic potential and their interactions with other constituents, an everlasting struggle of translating and evaluating the preclinical data in living organisms. As Greco et al. (1996) [33] pointed out in reference to anticancer agents, the therapeutic synergy is present when “the observed effect of the combination is more than what would be predicted from good knowledge of the effects of each agent working alone” and there is a fine lining between therapeutic synergy and in vitro synergy.

A therapeutic scheme of drugs must exhibit favorable outcomes at the lowest possible doses. However, when it comes to phytochemicals, this approach is also meaningful, with efficacy and toxicity being at the forefront of every intervention. The need to take the beneficial possibilities of polyphenol-rich interventions one step further in the management of challenging diseases such as OA is unequivocal. This may be achieved with better defined and standardized proprieties that combine constituents with proven therapeutic properties mediated by thoroughly elucidated molecular interactions.

In agreement with this course of action, a standardized extract from the bark of the French maritime pine (*Pinus pinaster Aiton*) Pycnogenol that consists of a mixture of polyphenols [34] has been extensively examined in different models. Preclinical investigation exhibited chondroprotective effects and inhibition of the transcription nuclear factor κB (NF-κB) [35] with the presence of the active ingredients of Pycnogenol not only in serum and blood cells but also in the synovial fluid of patients with severe OA [36]. Furthermore, a RCT showed that all three subscales of WOMAC—pain, stiffness, and physical function—decreased significantly in patients with mild OA receiving 3 × 50 mg of Pycnogenol daily for 3 months compared to the placebo group [37]. Two other RCTs have corroborated these results [38,39].

Another medical food, Flavocoxid (Limbrel), is a product of two flavonoids, baicalin and catechins, that are derived from the botanicals *Scutellaria baicalensis* and *Acacia catechu*, respectively [40]. In a 1-month pilot study, Flavocoxid was deemed as effective as naproxen in reducing short-form WOMAC, physician’s global assessment of disease activity (PGAD), subject’s global assessment of disease activity (SGAD), and subject’s global assessment of disease related discomfort (SGADc), with similar but high percentages of adverse effects for the two groups. In a larger cohort under the same setting but for a longer period, noninferiority of this propriety was demonstrated through the use of WOMAC [41]. At a molecular level, Flavocoxid exerts positive effects mainly through its implication on arachidonic acid metabolism [42,43,44]. Regarding its toxicity and association with acute liver injury and hypersensitivity pneumonitis, a large cohort recently demonstrated that the rate of such incidents was low and marginally elevated compared to NSAIDs [45].

In another RCT, researchers evaluated Reparagen, a polyherbal mixture of extracts from *Uncaria guianensis* (Amazonian tea) and *Lepidium meyenii* (Andean vegetable) against glucosamine, a conventional alternative to NSAIDs [46]. Whereas both treatments yielded significant results in the primary endpoint with a 20% reduction in WOMAC pain in a 2-month period, Raparagen managed to reduce the use of rescue medication.

## 5. Preclinical and Clinical Studies

The 3-year literature survey resulted in six publications that investigated the effect of combined polyphenols in OA. Their results are presented in Table 1.

### 5.1. Grapeseed and Olive Extracts

A 2016 study by Mevel et al. [47] which investigated a standardized combination of two extracts from grapeseeds and olive in two post-traumatic OA animal models (PTOA) paved the way for the evaluation of this extract at a clinical level by the same research team [48]. In the light of the promising preclinical results of the grapeseed and olive extract (OPCO), which has a high content of HT and procyanidins (PCy), the research team attempted illuminate the chemical modifications that polyphenols undergo in the human body and determine whether in vitro results could correlate with an ex vivo model. To this end, volunteers were administered 3.2 g OPCO extract, and serum samples were obtained. Next, human articular chondrocytes (HACs) were cotreated with IL-1β and the human serum. HACs showed a decrease of IL-1β-induced production of MMP-13, NO, and prostaglandin E_2_ (PGE_2_). The results indicated the absorption and availability of the OPCO constituents in humans as they were in agreement with the in vitro data model in which HACs were cotreated with IL-1β and olive extract (PHO), grape extract (OPC), or OPCO extract. Furthermore, the results demonstrated a decrease in NF-κB p65 subunit translocation and thus the blockade in NF-κB activation as the mechanism underlying the regulation of MMP-13, NO, and PGE_2_. The authors hypothesized that the effect of OPCO extract in HACs was not synergistic but cumulative with regard to the production of NO and PGE_2_.

### 5.2. Astragalus membranaceus and Lithospermum erythrorhizon Extracts (ALM16)

A recent study evaluated the synergistic effect of a mixture of two medicinal herbal extracts, *Astragalus membranaceus* and *Lithospermum erythrorhizon*, mixed at an optimal rate (ALM16) [49]. HPLC-DAD profiling revealed the active compounds of ALM16 to be calycosin (isoflavone), calycosin-7-O-β-D-glucosid,e and lithospermic acid (polycyclic phenolic carboxylic acid). The herbal mixture demonstrated significant chondroprotective effects in vitro as it reduced the IL-1β-induced production of MMP-1, -3, and -13 in a dose-dependent manner and decreased glycosaminoglycans (GAG) degradation. In vivo ALM16 significantly decreased the frequency of abdominal writhing in mice and increased the paw withdrawal threshold (PWT) in rats, suggesting antinociceptive properties. It also decreased paw edema and the arthritis score evaluated by histopathological examination. Regarding the synergistic phenomena—and while individual phytochemicals were not examined—ALM16 significantly decreased the MMP-13 activity, paw edema, and arthritis score and increased PWT more than individual extracts.

### 5.3. Terminalia chebula, Curcuma longa, and Boswellia serrata Extracts (LI73014F2)

Karlapudi and colleagues [50] evaluated a novel composition of *Terminalia chebula* fruit, *Curcuma longa* rhizome, and *Boswellia serrata* gum resin extracts (LI73014F2) in relation to knee OA. LI73014F2 is rich in gallic acid, ellagic acid, curcuminoids, and acetyl-11-keto-β-boswellic acid (AKBA). A composition of 2:1:2 exhibited the highest inhibition of 5-LOX enzyme. Thus, the ratio was used for the standardization of the capsules. Furthermore, the inhibitory effect of LI73014F2 on the enzyme activity was shown to be significantly higher in comparison with individual ingredients, suggesting an added effect due to synergism. LI73014F2 was evaluated in a preclinical monoiodoacetate (MIA)-induced OA rat model as well as in a clinical randomized trial. In particular, experimental rats receiving different doses of LI73014F2 demonstrated improvement in weight-bearing capacities and in pain perception in a degree comparable to the effect of tramadol. In the 3-month randomized, placebo-controlled, double-blinded study, not only the safety of the formula was demonstrated but also the statistically significant improvement in WOMAC, the Lequesne Functional Index (LFI), and VAS score in patients treated with 400 mg of LI73014F2 daily.

At the cellular level, a study by Kim et al. [51] verified the decrease of the enzymatic activity of COX-2 and 5-LOX as the levels of PGE_2_ and LTB4 were significantly reduced in IL-1β-stimulated HACs. Treatment of HACs with LI73014F2 also reduced the expression of inflammatory cytokines, exhibited chondroprotective properties, and reduced apoptotic biomarkers via the inhibition of the NF-κB/MAPK signaling pathways.

Similarly, in a MIA-induced OA rat model, treatment with LI73014F2 significantly limited the clinical manifestation of OA symptoms such as pain, swelling, and limping [52]. In this case, the inflammatory load was evaluated in the synovial fluid. Proinflammatory mediators and cytokines were reduced. Expression of MMPs was also reduced in the articular cartilage, and histological evaluation revealed improvement in structural morphological changes in the cartilage, synovial membrane, and fibrous tissue.

### 5.4. Combinations with Curcumin

D’Ascola and coworkers attempted to determine whether synergy takes place between curcumin and specific constituents such as Flavocoxid (a standardized mixture of baicalin and catechin) and β-caryophyllene (BCP) [53]. In this respect, they referred to the established work of Chou and Talalay that introduced the β-caryophyllene (BCP) theory and its computer software [32]. This theory is based on the median effect equation, which is visualized in the median effect plot. According to this theory, a potent synergy for a combination is evaluated with the combination index (CI) where values below 1 reflect synergy and values above or equal to 1 mean antagonism or an additive effect respectively. HACs were triggered with lipopolysaccharide (LPS) or with IL-1β and treated with curcumin, Flavocoxid, BCP alone, curcumin + Flavocoxid, or curcumin + BCP. Overall, the combinations reduced the expression of IL-1β, NF-κB, and STAT3 and restored the expression of collagen type II alpha 1 (COL2A1) more efficiently compared to Flavocoxid, BCP, and curcumin alone. Finally, the nature of the interactions in the aforementioned combinations was deemed to be synergistic.

### 5.5. Kaempferol and Apigenin

The effect of two flavonoids, kaempferol and apigenin, in combination with MSCs on anterior cruciate ligament transection (ACLT)-induced OA in rats, was investigated in a recent study [54]. After treatment of synovial membrane-derived MSCs (SMMSCs) with the two flavonoids for evaluation of cell viability, concentrations of 10 μM and 20 μM for kaempferol and 0.1 μM and 0.3 μM for apigenin were selected for the experiments.

Cartilage homogenate of OA rats receiving kaempferol (10 μM or 20 μM) with MSCs showed the greatest decrease in inflammatory cytokines TNF-α and IL-1β. The activity of the antioxidant superoxide dismutase (SOD) increased and reached the levels of sham group (healthy rats) with treatment of MSCs and the two flavonoids. Furthermore, the expression of IL-1β, TNF-α, and iNOS and the production of malondialdehyde (MDA) were significantly inhibited in the MSCs and kaempferol (20 μM) group. Cotreatment with MSCs, kaempferol, and apigenin significantly decreased the expression of MMP-3 and MMP-13 catabolic enzymes; increased the expression of aggrecan, COL2A1, and transcription factor SOX-9; and improved histopathologic scores. Taken together, these results indicate that the combination treatment of apigenin, kaempferol, and MSCs accelerates regeneration processes of the cartilage, decreases degradation of the cartilage and production of inflammatory cytokines, increases the antioxidant status of the implicated tissues, and limits the consequent oxidation of cell components.

### 5.6. Turmeric Extract, Black Pepper, and Ginger

Turmeric extract, ginger (gingerol), and black pepper (pyrene) in the form of a dietary supplement called Mixodin were evaluated in a randomized, double-blinded, controlled, clinical trial that lasted 1 month [55]. Overall, 60 participants with moderate knee OA (Kellgren and Lawrence: 2, 3) were allocated into 2 groups: 30 subjects received Naproxen, a NSAID, and 30 received the herbal formulation. Dietary intake was recorded through 24-hour recalls in order to ensure the stable intake of dietary phytochemicals, with the Naproxen group being advised to refrain from consuming the constituents of the supplement. Serum levels of the inflammatory mediator PGE_2_ were determined before and after the treatment, and the results showed a similar decrease for both groups that reached statistical significance (*p* < 0.001). The nature of the interactions was not clarified as synergistic but the results pose a promising alternative to the use of NSAIDs.

## 6. Conclusive Remarks and Future Perspectives

Synergy derives from the Greek word synergos (syn—“together” and ergo—“work”), meaning working together. Synergistic interactions between phytochemicals in whole foods are a well-studied factuality [56]. Thus, one can argue that their unique combinations in foods possess synergistic/additive attributes that encapsulated extracts from herbal sources and purified phytochemicals cannot mimic [57]. However, nutrition alone, even when rich in fruits and vegetables, cannot attain sufficient concentrations of these compounds. Higher pharmacological doses of such constituents are required in the treatment of multifactorial diseases. As researchers attain an in-depth knowledge of the bioavailability and the chemical modifications that polyphenols undergo, more options are presented regarding how to employ these constituents.

However, a skepticism that arises in the case of synergism is the evaluation and quantification of such results in a clinically relevant way as the very nature of interactions is elusive to begin with. Consequently, an important step in this venture is the establishment of the terminology used to define interactions, as well as the model and statistical approach used to quantify those interactions [58].

Loewe additivity constitutes a cornerstone for the quantification of synergistic interactions as it represents a reference model that establishes the “null hypothesis,” i.e., an interaction devoid of synergistic or antagonistic relationships [59] expanded by Chou and Talalay [32] and the use of CI. Loewe additivity and most models make assumptions that are imperative to form the basis of models, but they predispose them with biases that derive from these assumptions. In combination with lack of consensus in the tools used [60], this makes it difficult to draw definite conclusions on the nature of the examined interactions.

The studies discussed in this review have evaluated combinations of extracts rich in polyphenols and different combinations of specific polyphenols in in vitro, ex vivo, and in vivo OA models. The evaluation of the type of the relationships between various combinations was mostly performed using t-tests and *p*-values. Only one study by D’Ascola and colleagues [53] utilized the CI, but even this approach entails pitfalls [61].

Another important parameter to consider when investigating the potential synergistic effects of phenolics in OA is the need for a priori knowledge of the molecular signaling pathways affected by the use of individual phenolic components. The studies analyzed in this review have confirmed the inhibition of Nf-κB and MAPK signaling pathways by curcumin-containing formulations [53,55]. Individually, curcumin can also induce autophagy, thus protecting the chondrocytes from apoptosis via the inhibition of the MAPK/ERK1/2 signaling pathway [62] and reducing inflammation by blocking the activation of TLR4/MyD88/NF-κB signaling pathway [63].

**Figure 1:**
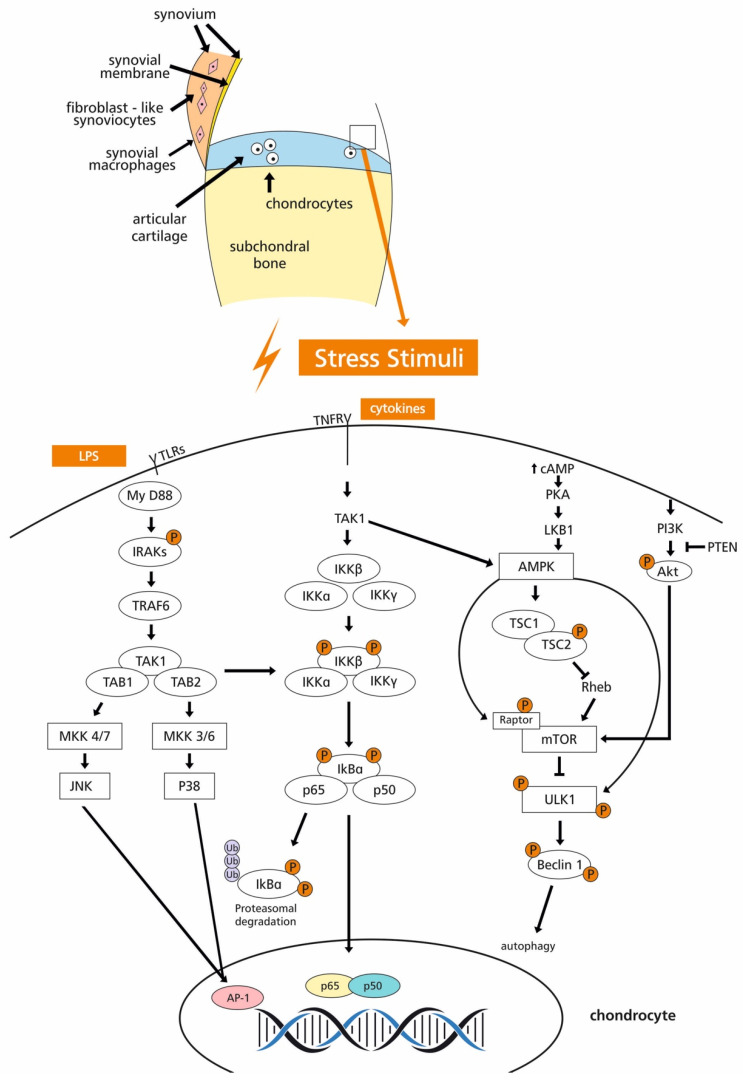
Some signal transduction pathways and their crosstalk in OA modulated by phenolic compounds comprising potent therapeutic targets. Pathways presented from left to right: TLR4 myD88 pathway (IRAKs: Interleukin-1 receptor-associated kinases, TRAF: TNFR-associated factor, TAK1: TGF-β-activated kinase 1, TAB: TAK1-binding proteins, MKK: mitogen-activated protein kinase kinase, JNK: c-Jun N-terminal kinase), canonical NF-κB pathway (TNFR: Tumor necrosis factor receptor, IKK: IκB kinase, IκB: NF-Κb protein), AMPK autophagy pathway (cAMP: Cyclic adenosine monophosphate, PKA: Protein kinase A, LKB1: Liver kinase B1, AMPK: AMP-activated protein kinase, TSC: Tuberous sclerosis complex, mTOR: Mammalian target of rapamycin, ULK1: Mammalian homolog of yeast Atg1), PI3K/Akt/mTOR pathway (PI3K: Phosphatidylinositol 3-kinase, Akt or PKB: Protein kinase B, PTEN: Phosphatase and tensin homolog deleted on chromosome 10).

Over the last few years, a number of polyphenols have been investigated regarding the mechanisms underlying their effects on OA (Figure 1). It has been shown that HT can regulate sirtuin (SIRT)-6-mediated autophagy in chondrocytes [64] and oleuropein can suppress the NF-κB and MAPK signaling pathways [65,66]. Resveratrol is another polyphenol that promotes chondrocyte autophagy via regulation of the regulating AMPK/mTOR signaling pathway [67]. It also prevents chondrocyte apoptosis via activation of the PI3K/Akt signaling pathway [68] and suppresses catabolic and inflammatory mediators via inhibition of both TLR4/MyD88-dependent and -independent signaling pathways [69]. Quercetin, a flavonol that can selectively clear senescent cells, has the potency of a senolytic drug [70]. Quercetin can induce the M2 polarization of macrophages [71] and relieve endoplasmic reticulum stress and chondrocyte apoptosis by activating the sirtuin1/adenosine monophosphate-activated protein kinase (SIRT1/AMPK) signaling pathway [72], decrease the production of proinflammatory cytokines by inhibiting the TLR-4/NF-κB pathway [73], and contribute to the physiological functions of mitochondria by upregulating the AMPK/SIRT1 signaling pathway [74].

**Figure 2:**
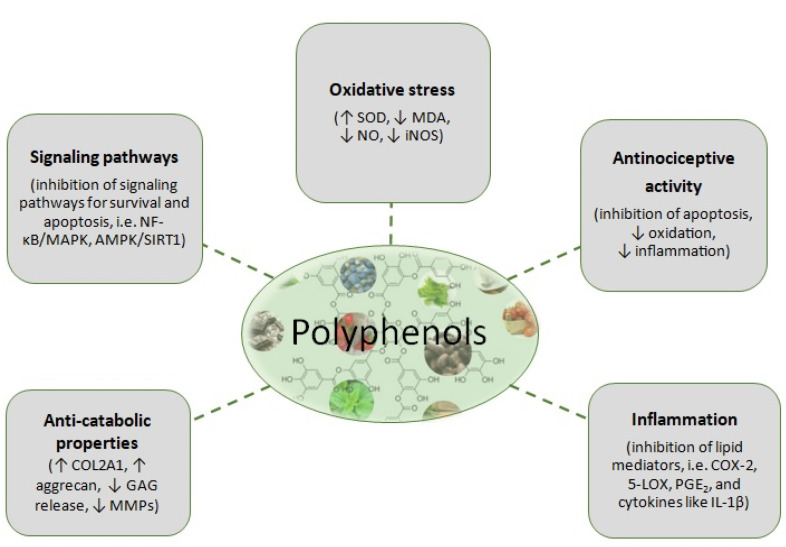
Putative mechanisms of action of polyphenols against OA. SOD: Superoxide dismutase, MDA: Malondialdehyde, NO: Nitric oxide, iNOS: Inducible nitric oxide synthase, COL2A1: Collagen type II alpha 1; GAG: Glycosaminoglycans, MMPs: Matrix metalloproteinases, COX-2: Cyclooxygenase-2, 5-LOX: 5-lipoxygenase, PGE_2_: Prostaglandin E_2_, IL-1β: Interleykin-1β, NF-κΒ: Nuclear factor kappa-Β.

A plethora of studies have advocated that a tendency is forming toward approaches that explore and seek to elucidate the interplay of high-value molecules such as polyphenols in inflammatory, oxidative diseases. OA poses a very challenging disease. To this point, results from numerous preclinical studies have indicated that polyphenols exert beneficial effects in OA with different potential underlying mechanisms (Figure 2). However, more clinical trials are required to evaluate not only the self-reported questionnaires of pain and physical function but also the biomarkers of inflammation and OS. Then, meaningful correlations between the subjective information obtained through the questionnaires and the objective obtained through serum and plasma evaluation of OA-related markers will be explored. This approach will enable a better understanding and clarification of the action of the polyphenols in in vitro models and the subsequent extrapolation of these data in clinical trials.

**Table 1:** Preclinical and clinical studies assessing the effect of synthetized extracts/polyphenols in osteoarthritis (OA).

First Author, Year	Composition of Pharmaceuticals	Experimental Design	Results
**Grapeseed and olive extract (OPCO)**			
Wauquier et al. 2019 [48] (in vitro, ex vivo)	(1) PHO extract (Olivex^®^, Grap’sud, Cruviers-Lascours, France), derived from olive, 6.5% of hydroxytyrosol/21% total polyphenol content; (2) OPC extract (Exgrape^®^ SEED, Grap’sud, Cruviers-Lascours, France), derived from grape seed, 30% of procyanidines/90% total polyphenol content; and (3) OPCO extract (Oleogrape^®^ SEED, Grap’sud, Cruviers-Lascours, France), derived from grapeseed and olive, 6.5% of hydroxytyrosol, 30% of procyanidines/90% total polyphenol content	HACs pretreated with grape extract, olive extract, or OPCO (10 µg/mL) for 24 h and then cotreated with IL-1β (1 ng/mL) for 24 h Fasted volunteers (*n* = 20) received 3.2 g of OPCO extract. Enriched serum with OPCO metabolites was collected at 100 min postingestion (peak absorption) HACs pretreated with 10% of human-enriched serum and then cotreated with IL-1β (1 ng/mL) for 24 h	Treatment with OPCO extract: 🖗IL-1β induced production of MMP-13, NO, PGE_2_ (anti-inflammatory effect) 🖗IL-1β mediated activation of the NF-κB p65 signaling pathway by reducing p65 translocation to the nucleus Treatment with human-enriched sera: 🖗IL-1β induced production of MMP-13, NO, PGE_2_
**Astragalus membranaceus and Lithospermum erythrorhizon extracts (ALM16)**			
Choi et al. 2019 [49] (in vitro, in vivo)	*A.membranaceus* and *L. erythrorhizon* extracts powder mixed together at a ratio of 7:3 (*w*/*w*) to prepare the final extract mixture (ALM16). Active compounds of ALM16 as quantified by HPLC-DAD were calycosin (0.571 mg/g), calycosin-7-O-β-D-glucoside (0.809 mg/g), and lithospermic acid (0.168 mg/g).	Human SW1353 chondrosarcoma cells pretreated with *A. membranaceus* extract (A), *L. erythrorhizon* extract (L) and a herbal mixture (7:3) of ethanol extracts of A & L (ALM16) (0, 25, 50, 100, 200 μg/mL) for 30 min and co-treated with IL-1β (20 ng/mL) for 24 h Male ICR mice (5 groups, *n* = 8/group) were administered: A (400 mg/kg b.w.), L (400 mg/kg b.w.), ALM16 (100, 200 and 400 mg/kg b.w.), or celecoxib™ (100 mg/kg b.w.) 1 h prior to the acetic acid injection MIA-injected rats (5 groups, *n* = 8/group) were treated once daily for 14 days with: 1. Normal, 2. control, 3. JOINS™ (extracts of oriental herbs as a positive control, 200 mg/kg b.w.), 4. A extract (400 mg/kg b.w.), 5. L extract (400 mg/kg b.w.), and 6–8. ALM16 (100, 200 and 400 mg/kg b.w., respectively)	In cells treated with ALM16: 🖗IL-1β induced production of MMPs (−1, −3 and −13) in a dose-dependent manner 🖗IL-1β induced GAG releasing In mice receiving ALM16: 🖗 Acetic acid-induced writhing response in mice (anti-analgesic effect) 🖗Carrageenan-induced paw edema in mice in ALM16 group in a dose-dependent manner (anti-edematous effect) In rats receiving ALM16: 🖞Paw withdrawal thresholds in MIA-induced OA rats in ALM16 group (change of mechanical allodynia) 🖗Histopathological changes (arthritis score) in MIA induced OA rats in 200 mg/kg ALM16 group
**Terminalia chebula, Curcuma longa, and Boswellia serrata extracts (LI73014F2)**			
Karlapudi et al. 2018 [50] (in vivo)	LI73014F2 comprises of the aqueous extract of T. chebula fruit and the alcohol extract of C. longa rhizome, and B. serrata extract at 2:1:2 ratio. Through HPLC, the major components were identified to be 1% gallic acid, 0.5% ellagic acid, 2.0% total curcuminoids, and 0.6% AKBA. For the purpose of the study, LI73014F2 was encapsulated in hard gelatin capsules with excipients microcrystalline cellulose powder (8%) and syloid (2%).	MIA induced OA in Sprague Dawley rats (*n* = 30, 5 groups): 1. Normal control, 2. MIA induced, 3. LI73014F2 (250 mg/kg), 4. LI73014F2 (500 mg/kg), 5. tramadol hydrochloride (10 mg/kg), 28 days A 90-day RCT, *n* = 105 randomized into 3 groups: Placebo (*n* = 35), 200 mg/day LI73014F2 (*n* = 35), 400 mg/day LI73014F2 (*n* = 35)	In rats receiving LI73014F2: 🖞Weight-bearing capacity (pain relief) 🖗Thermal hyperalgesia (pain perception) In humans receiving LI73014F2: 🖗WOMAC scores (pain, stiffness, physical function), VAS scores, LFI
Kim et al. 2020, a [51]	LI73014F2 and the individual extracts were obtained from Laila Nutraceuticals	HACs cotreated with individual extracts of T. chebula (TCE; 50 μg/mL), C. longa (CLE; 50 μg/mL), B. serrata (BSE; 50 μg/mL), LI73014F2 (50 µg/mL) and IL-1β (10 ng/mL) for 24 h HACs co-treated with LI73014F2 (0, 12.5, 25, 50 μg/mL) and IL-1β (10 ng/mL) for 24 h HACs pretreated with LI73014F2 (12.5, 25, 50 μg/mL) for 24 h and then co-treated IL-1β (10 ng/mL) for 30 min	In LI73014F2 treated chondrocytes: 🖗IL-1β induced expression of COX-2, mPGES-1, PGE_2_, 5-LOX and LTB_4_ 🖗IL-1β induced expression of TNF-α, IL-6 🖗IL-1β induced production of MMP-2, -3, -9, -13 🖗IL-1β induced expression of Bax/Bcl-2, caspase-3,-9, PARP (apoptotic markers) 🖗IL-1β induced phosphorylation of NF-κB p65 and p38 MAPK
Kim et al. 2020, b [52]	Through HPLC, the composition of LI73014F2 was determined to be gallic acid (18 mg/g), total curcuminoids (35 mg/g), and AKBA (9 mg/g).	MIA induced OA in Sprague Dawley rats (6 groups, *n* = 8 rats/group); 1. control + vehicle, 2. MIA-induced control + vehicle, 3. MIA + 25 mg/kg LI73014F2, 4. MIA + 50 mg/kg LI73014F2, 5. MIA + 100 mg/kg LI73014F2, 6. MIA + 20 mg/kg ibuprofen for 21 days	In LI73014F2 groups: 🖞Weight-bearing capabilities (pain relief) 🖗Arthritis index In synovial fluid from LI73014F2 groups: 🖗Production of IL-1β In articular cartilage from LI73014F2 groups: 🖗Expression of IL-1β, IL-6, TNF-α, COX-2, 5-LOX, PGE_2_, LTB_4_, MMP-2, -3, -9 🖗Mankin score (structural OA related morphological changes)
**Curcumin+**			
D’Ascola et al. 2019 [53] (in vitro)	Flavocoxid, a FDA-regulated medical food known as Limbrel^®^ of Primus Pharmaceuticals, is a mixed extract of *Scutellaria baicalensis* and *Acacia catechu* rich in the flavonoids baicalin and catechin	HACs treated with LPS (2 μg/mL) or with IL-1β (10 ng/mL) alone or in combination with curcumin (0.65, 1.25, 2.5, 5, and 10 μg/mL) and with flavocoxid (4, 8, 16, 32, and 64 μg/mL) HACs treated with LPS (2 μg/mL) or with IL-1β (10 ng/mL) alone or in combination with curcumin at the same doses reported above and with BCP (1.25, 2.5, 5, 10, or 20 μg/mL)	Curcumin + Flavocoxid, Curcumin + BCP 🖗LPS induced expression of NF-κB and STAT3 mRNA 🖗IL-1β or LPS induced decrease of expression of COL2A1
**Kaempferol & Apigenin**			
Estakhri et al. 2020 [54] (in vivo)	-	ACTL-induced OA in Sprague Dawley rats (6 groups, *n* = 10/group); 1. Νο ACLT, 2. ACTL (Negative control), 3. Positive control: ACLT + treatment with Hyaluronic acid (H), 4. OA + treatment with MSCs, 5. OA + kaempferol (K) 10 μM, 6. OA + MSCs and K 10 μM, 7. OA + K 20 μM, 8. OA + MSCs and K 20 μM, 9. OA + K 10 μM + apigenin (A) 0.1 μM, 10. OA + MSCs + K 10 μM + A 0.1 μM	Greatest results in cartilage homogenate: 🖗IL-1β and TNF-α in OA + MSCs + K 10 and 20 μM groups 🖞SOD in OA + MSCs + K 10 μM and K 20 μM, OA + K 20 μM and OA + A 0.1 μM + K 10 μM groups 🖗OA score as seen in radiographs in OA + H, OA + MSCs and OA + MSCs + K 10 μM groups Improvement in histopathologic scores in OA + MSCs and OA + MSCs + K + A groups 🖗safranin O staining score in OA + MSCs + K + A group 🖗MDA in OA + MSCs + K 10 μM and K20 μM and OA + MSCs + K + A groups 🖗IL-1β, TNF-α, iNOS, MMP-3, -13 expression in OA + MSCs + K 20 μM and OA + MSCs + K + A groups 🖞COL2A1, SOX-9, aggrecan expression in OA + MSCs + K + A group
**Turmeric extract, black pepper, and ginger**			
Heidari-Beni et al. 2020 [55] In vivo (clinical)	Mixodin is a dietary supplement that consists of turmeric extract (curcumin; 300 mg/g), ginger extract (gingerol; 7.5 mg/g), and black pepper extract (piperine; 3.75 mg/g).	A randomized, double-blinded, controlled clinical trial, 60 volunteers with mild knee OA received Mixodin (*n* = 30) which contained curcumin (300 mg), gingerols (7.5 mg) and piperine (3.75 mg) or Naproxen (*n* = 30)for 4 weeks twice a day after meals	🖗PGE_2_ in both groups

## Data Availability

No new data were created or analyzed in this study. Data sharing is not applicable to this article.

## Abbreviations

ACLT	cruciate ligament transection
ACR	American College of Rheumatology
ACs	articular condrocytes
ADAMTS	disintegrin and metalloprotease with thrombospondin motifs
BCP	β-caryophyllene
CI	combination index
COL2A1	collagen type II alpha 1
COX-2	cyclooxygenase-2
DAMPs	damage associated molecular patterns
ECM	extracellular matrix
EGCG	epigallocatechin gallate
GAG	glycosaminoglycans
HACs	human articular chondrocytes
HT	hydroxytyrosol
iNOS	inducible nitric oxide synthase
LFI	Lequesne Functional Index
5-LOX	5-lipoxygenase
LPFI	Lequesne’s Pain-Function Index
LPS	lipopolysaccharide
MDA	malondialdehyde
MIA	monoiodoacetate
MMPs	matrix metalloproteinases
MSCs	mesenchymal stem cells
MyD88	myeloid differentiation factor 88
NF-κΒ	nuclear factor kappa-Β
NLRs	NOD-like receptors
NO	nitric oxide
NSAIDs	nonsteroidal anti-inflammatory drugs
OA	osteoarthritis
OARSI	Osteoarthritis Research Society International
OPC	grape extract
OPCO	grape seed and olive extract
OS	oxidative stress
PCy	procyanidins
PGA	patient’s global assessment
PGAD	physician’s global assessment of disease activity
PGE_2_	prostaglandin E_2_
PHO	olive extract
PRRs	pattern recognition receptors
PTOA	post-traumatic OA animal models
PWT	paw withdrawal threshold
RCT	randomized controlled trial
ROS	reactive oxygen species
RosA	rosmarinic acid
SGAD	subject’s global assessment of disease activity
SGADc	subject’s global assessment of disease related discomfort
SIRT1/AMPK	sirtuin1/adenosine monophosphate-activated protein kinase
SMMSCs	synovial membrane-derived mesenchymal stem cells
SOD	superoxide dismoutase
TLRs	toll-like receptors
TRIF	toll/interferon response factor
VAS	Visual Analog Scale
WOMAC	Western Ontario and McMaster Universities Osteoarthritis Index
